# Supplementary figures and images for: Propagation of orientation selectivity in a spiking network model of layered primary visual cortex
Source: PLoS Comput Biol. 2019 Jul 19;15(7):e1007080. doi: 10.1371/journal.pcbi.1007080 (PMC6641049; doi:10.1371/journal.pcbi.1007080)

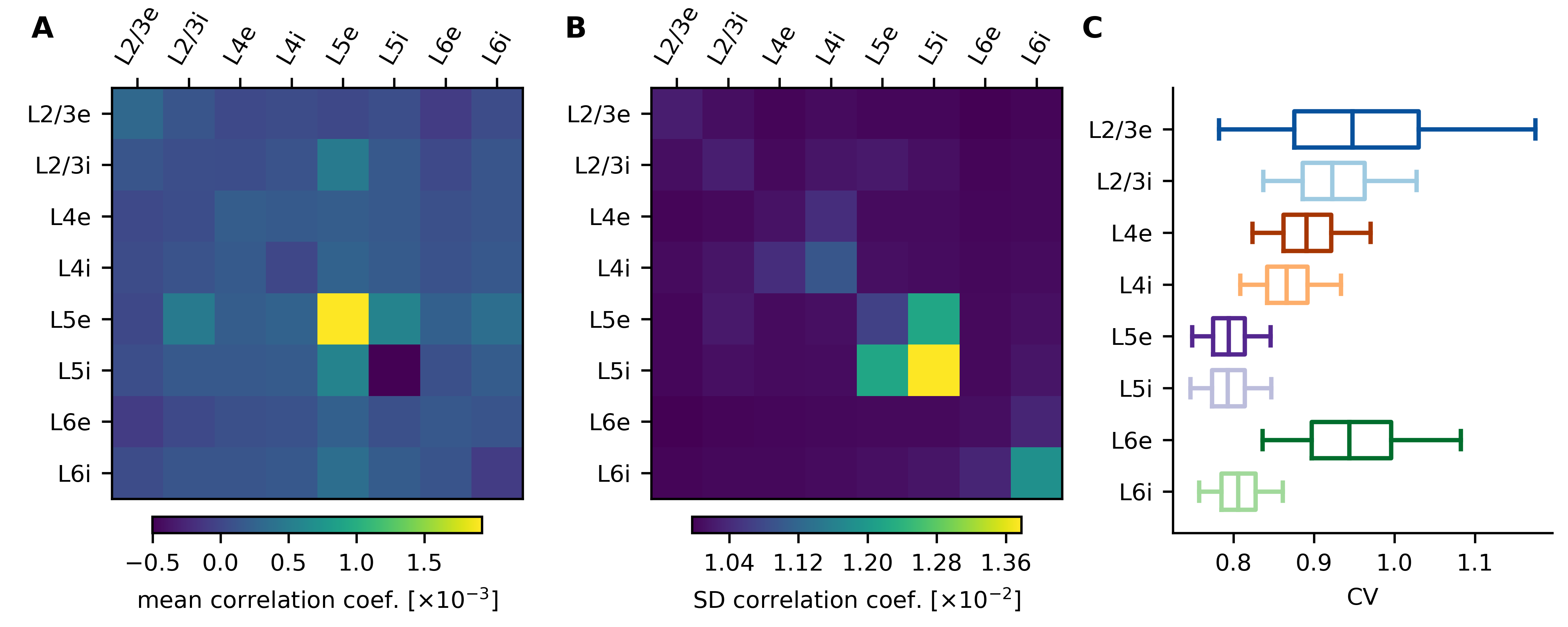

Supplement: S1 Fig — A-B Mean and standard deviation of spike count correlations between neurons from different populations. A bin size of 10 ms was used. C Distributions of the coefficient of variation of inter-spike-intervals for neurons in different populations. Same quantiles as described in Fig 2 are shown. (TIF) [file pcbi.1007080.s001.tif]

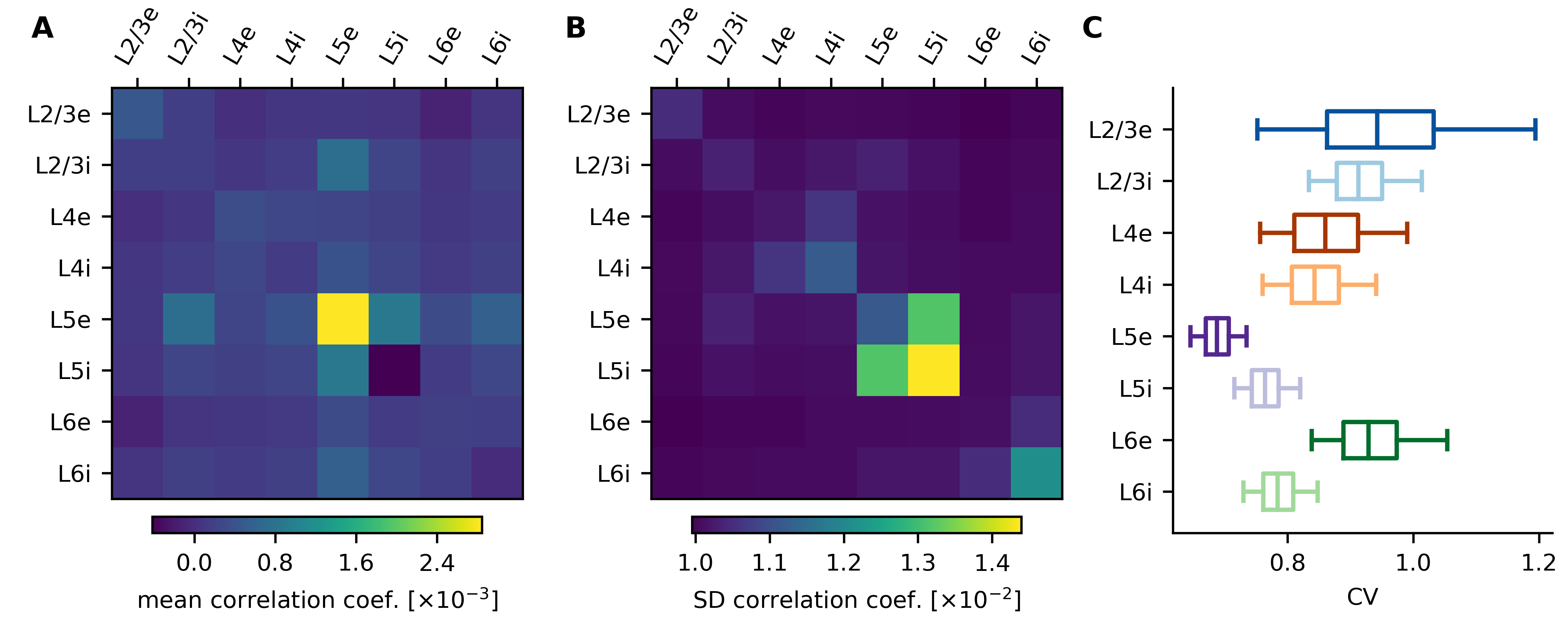

Supplement: S2 Fig — A-B Mean and standard deviation of spike count correlations between neurons from different populations. A bin size of 10 ms was used. C Distributions of the coefficient of variation of inter-spike-intervals for neurons in different populations. Same quantiles as described in Fig 2 are shown. (TIF) [file pcbi.1007080.s002.tif]

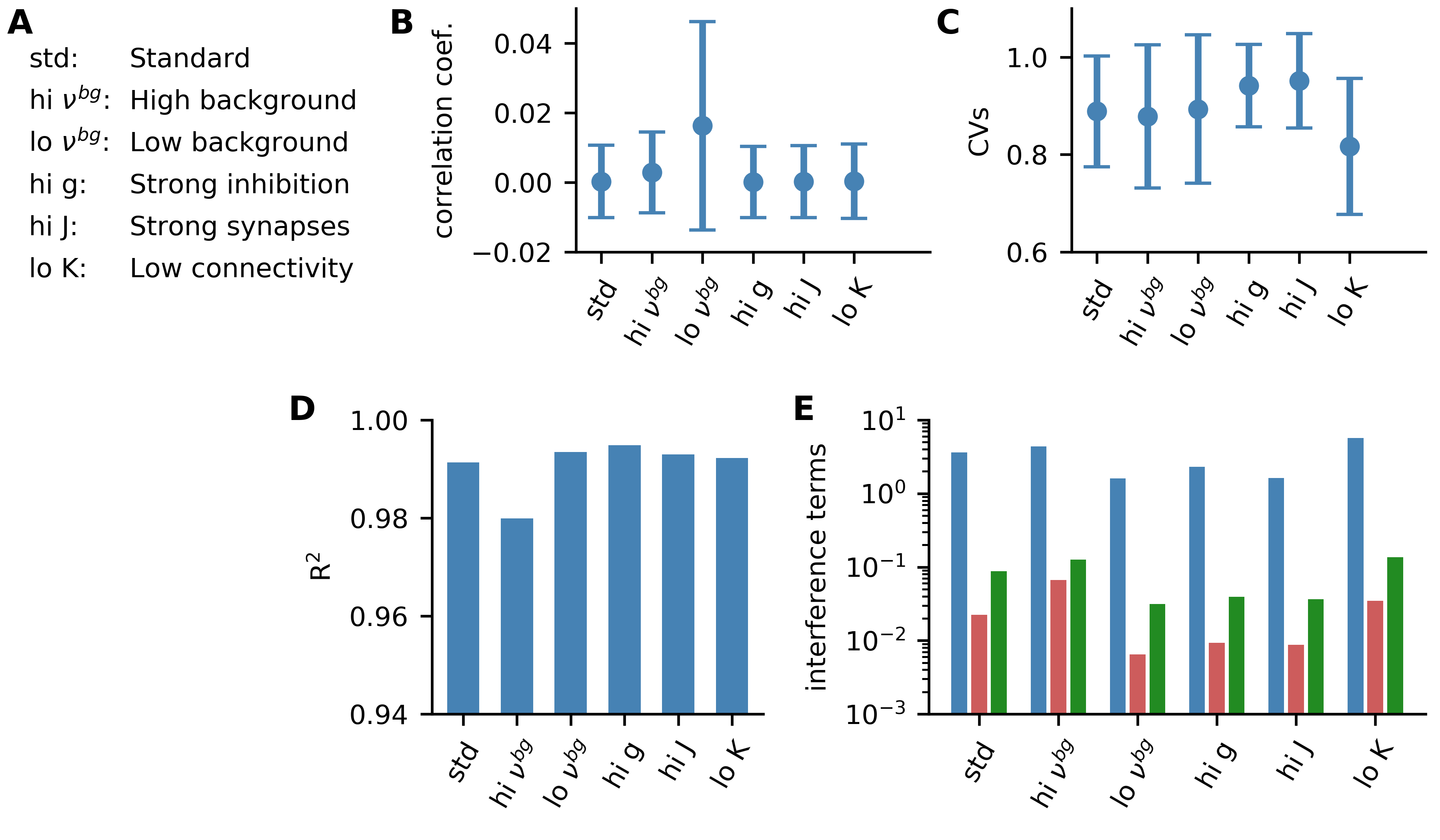

Supplement: S3 Fig — A Abbreviations used in other panels. B Mean and SD of correlation coefficient over all populations. C Mean and SD of CV of inter-spike-intervals over all populations. D Coefficient of determination (R2) quantifying the fit between a direct linear solution and a baseline and modulation decomposition. E Magnitudes of interference terms in Eq 12 on logarithmic scale (blue: Δβ, red: QΔνM, green: SΔνB). (TIF) [file pcbi.1007080.s003.tif]

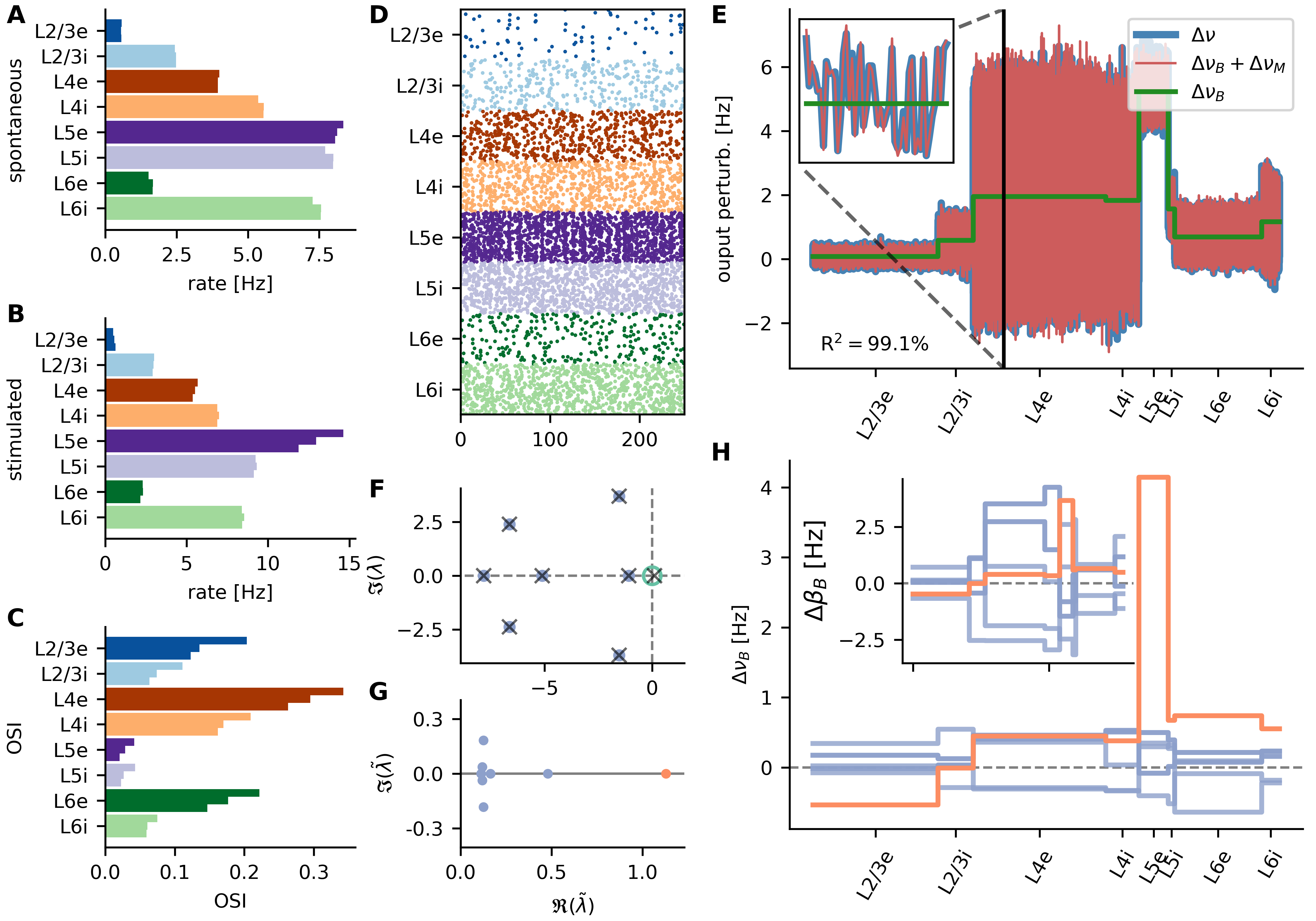

Supplement: S4 Fig — A/B Mean firing rates for spontaneous and stimulated condition. For each population, the results of the spiking model (Model A, top), nonlinear rate model (Model B, middle) and linear model (Model C, bottom) are shown. C Mean orientation selectivities for stimulated condition for all three models. D Raster plot for the stimulated condition for 250 ms of 500 neurons in each population. E Comparison of baseline and modulation solution with direct solution of the linear model (cf. Fig 6C). F Eigenvalue spectrum of the effective connectivity W (cf. Fig 7B). G Input perturbation ΔβB (inset) and output perturbation ΔνB decomposed into input and output modes, respectively (cf. Fig 7D). (TIF) [file pcbi.1007080.s004.tif]

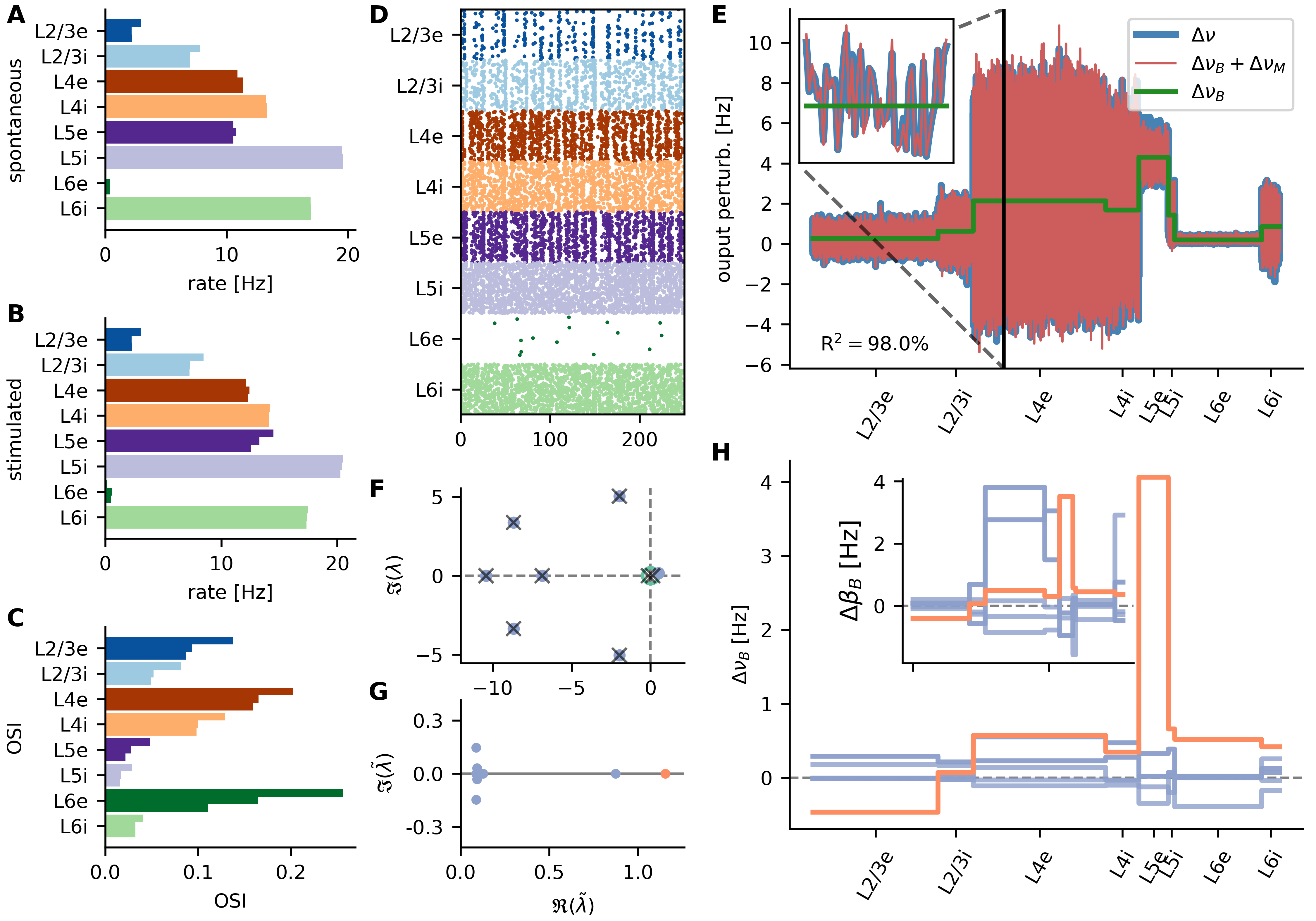

Supplement: S5 Fig — A/B Mean firing rates for spontaneous and stimulated condition. For each population, the results of the spiking model (Model A, top), nonlinear rate model (Model B, middle) and linear model (Model C, bottom) are shown. C Mean orientation selectivities for stimulated condition for all three models. D Raster plot for the stimulated condition for 250 ms of 500 neurons in each population. E Comparison of baseline and modulation solution with direct solution of the linear model (cf. Fig 6C). F Eigenvalue spectrum of the effective connectivity W (cf. Fig 7B). G Input perturbation ΔβB (inset) and output perturbation ΔνB decomposed into input and output modes, respectively (cf. Fig 7D). (TIF) [file pcbi.1007080.s005.tif]

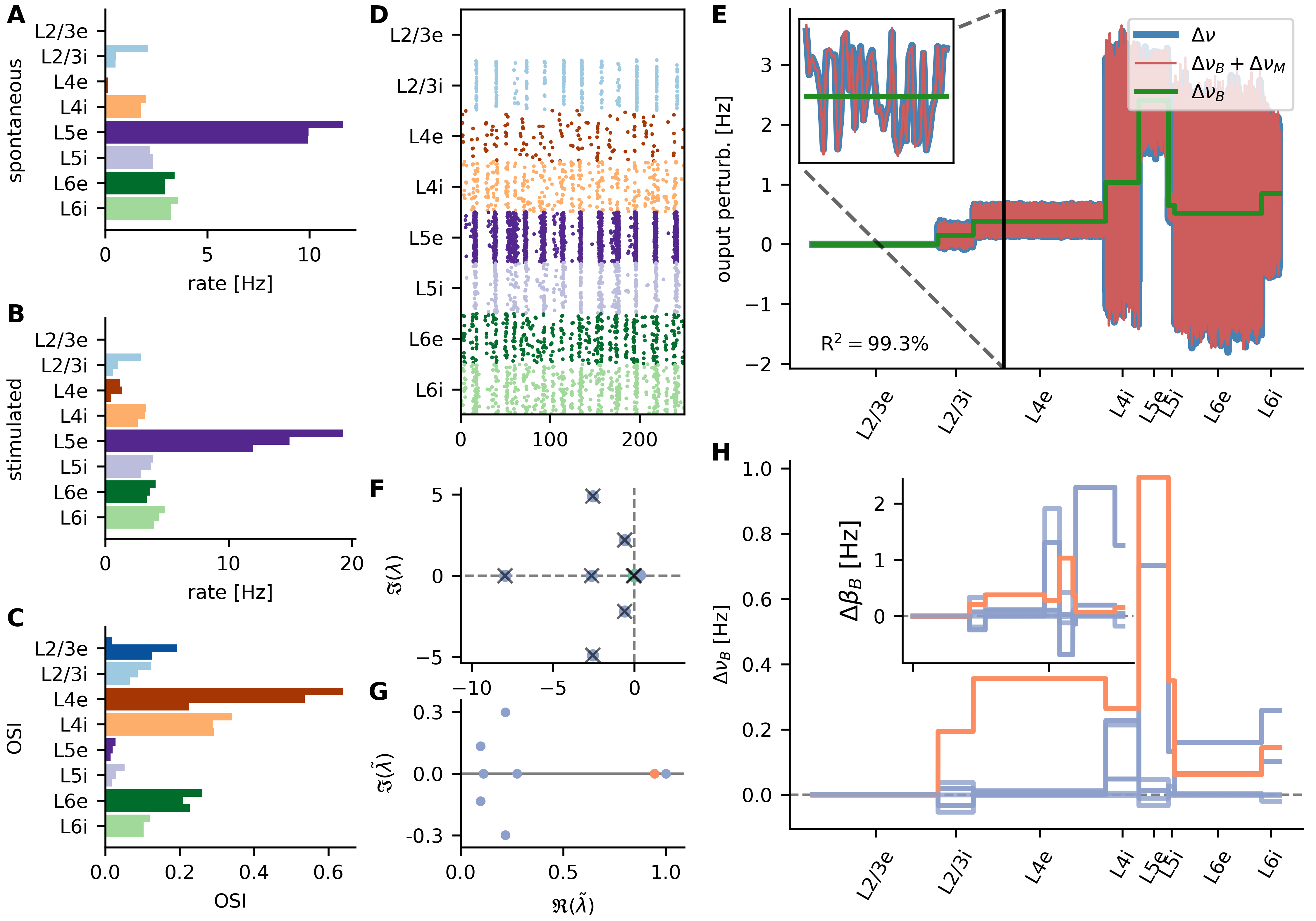

Supplement: S6 Fig — A/B Mean firing rates for spontaneous and stimulated condition. For each population, the results of the spiking model (Model A, top), nonlinear rate model (Model B, middle) and linear model (Model C, bottom) are shown. C Mean orientation selectivities for stimulated condition for all three models. D Raster plot for the stimulated condition for 250 ms of 500 neurons in each population. E Comparison of baseline and modulation solution with direct solution of the linear model (cf. Fig 6C). F Eigenvalue spectrum of the effective connectivity W (cf. Fig 7B). G Input perturbation ΔβB (inset) and output perturbation ΔνB decomposed into input and output modes, respectively (cf. Fig 7D). (TIF) [file pcbi.1007080.s006.tif]

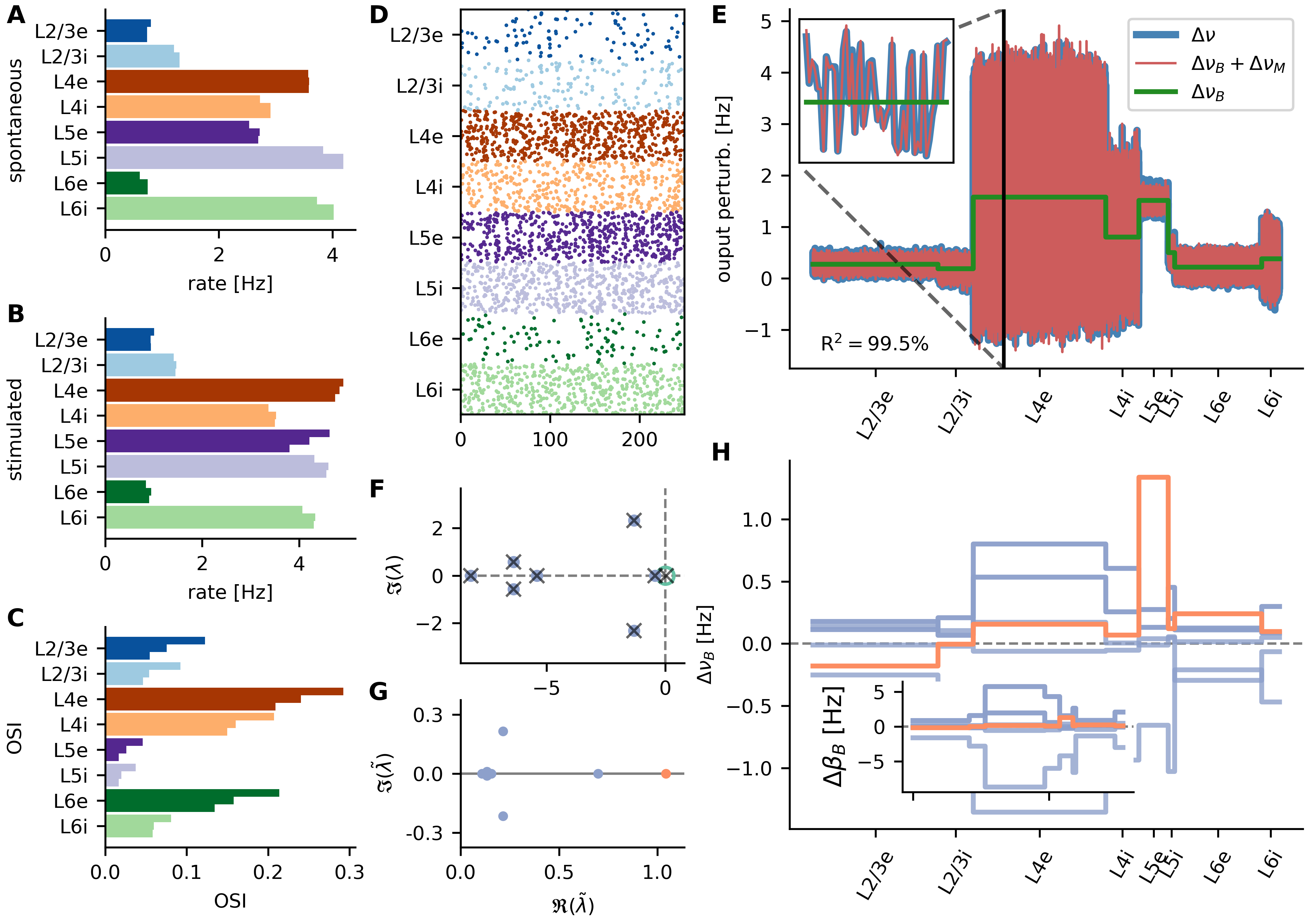

Supplement: S7 Fig — A/B Mean firing rates for spontaneous and stimulated condition. For each population, the results of the spiking model (Model A, top), nonlinear rate model (Model B, middle) and linear model (Model C, bottom) are shown. C Mean orientation selectivities for stimulated condition for all three models. D Raster plot for the stimulated condition for 250 ms of 500 neurons in each population. E Comparison of baseline and modulation solution with direct solution of the linear model (cf. Fig 6C). F Eigenvalue spectrum of the effective connectivity W (cf. Fig 7B). G Input perturbation ΔβB (inset) and output perturbation ΔνB decomposed into input and output modes, respectively (cf. Fig 7D). (TIF) [file pcbi.1007080.s007.tif]

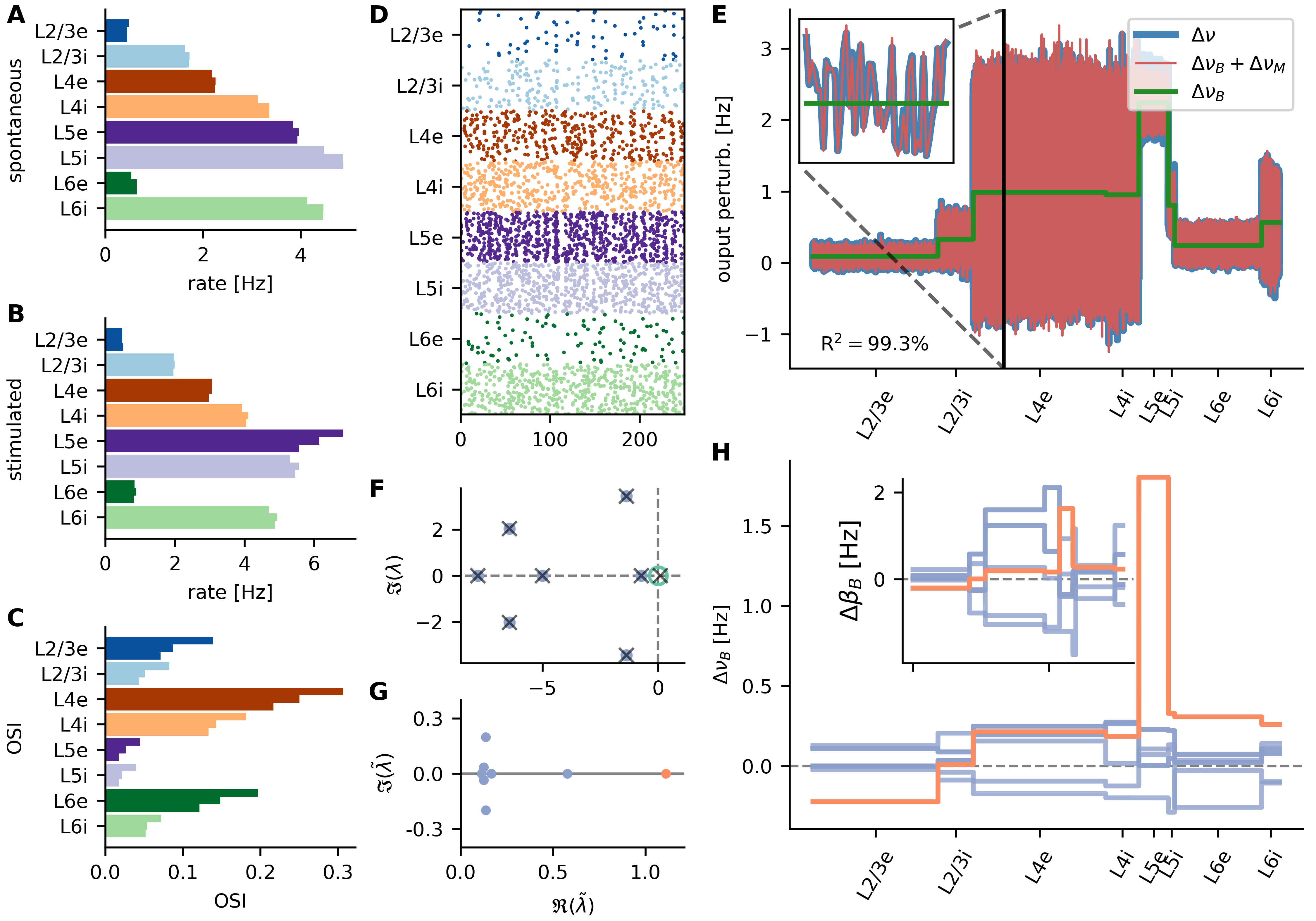

Supplement: S8 Fig — A/B Mean firing rates for spontaneous and stimulated condition. For each population, the results of the spiking model (Model A, top), nonlinear rate model (Model B, middle) and linear model (Model C, bottom) are shown. C Mean orientation selectivities for stimulated condition for all three models. D Raster plot for the stimulated condition for 250 ms of 500 neurons in each population. E Comparison of baseline and modulation solution with direct solution of the linear model (cf. Fig 6C). F Eigenvalue spectrum of the effective connectivity W (cf. Fig 7B). G Input perturbation ΔβB (inset) and output perturbation ΔνB decomposed into input and output modes, respectively (cf. Fig 7D). (TIF) [file pcbi.1007080.s008.tif]

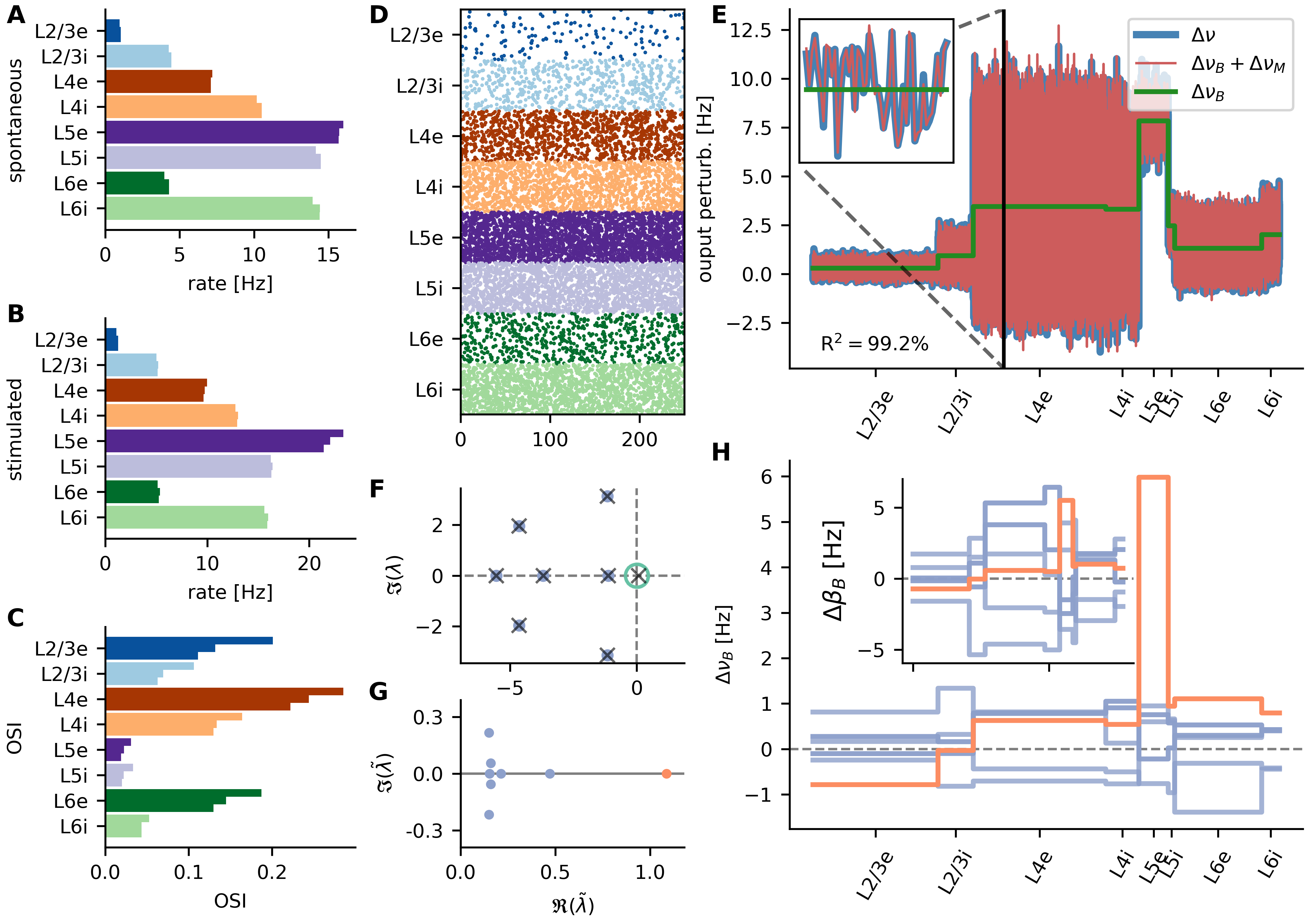

Supplement: S9 Fig — A/B Mean firing rates for spontaneous and stimulated condition. For each population, the results of the spiking model (Model A, top), nonlinear rate model (Model B, middle) and linear model (Model C, bottom) are shown. C Mean orientation selectivities for stimulated condition for all three models. D Raster plot for the stimulated condition for 250 ms of 500 neurons in each population. E Comparison of baseline and modulation solution with direct solution of the linear model (cf. Fig 6C). F Eigenvalue spectrum of the effective connectivity W (cf. Fig 7B). G Input perturbation ΔβB (inset) and output perturbation ΔνB decomposed into input and output modes, respectively (cf. Fig 7D). (TIF) [file pcbi.1007080.s009.tif]

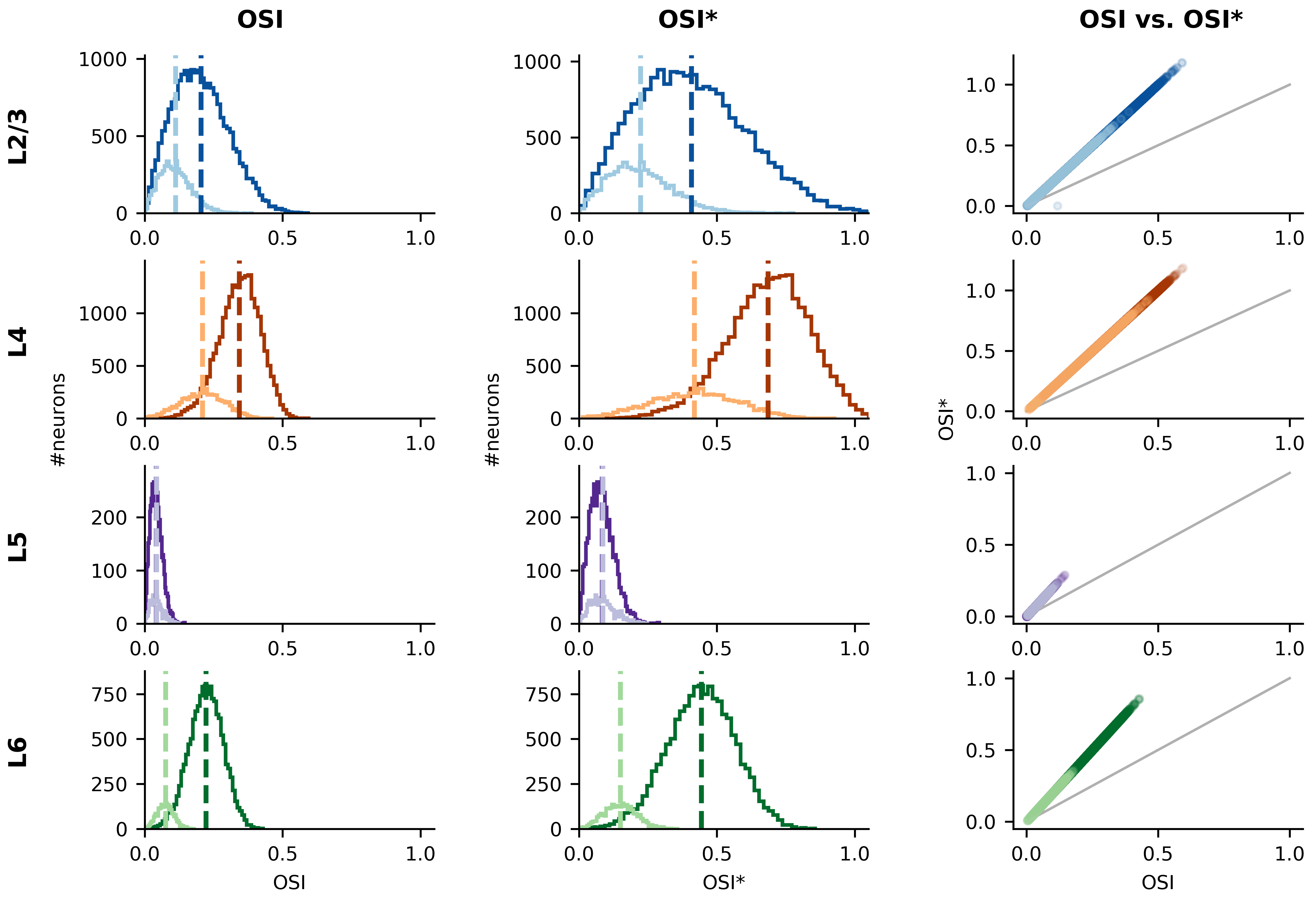

Supplement: S10 Fig — Left column: Distribution of orientation selectivity measure based on circular statistics (OSI). Middle column: Orientation selectivity measure based on cosine fit (OSI*). Right column: Comparison of the two orientation selectivity measures. Each dot represents a single neuron in the respective population. (TIF) [file pcbi.1007080.s010.tif]

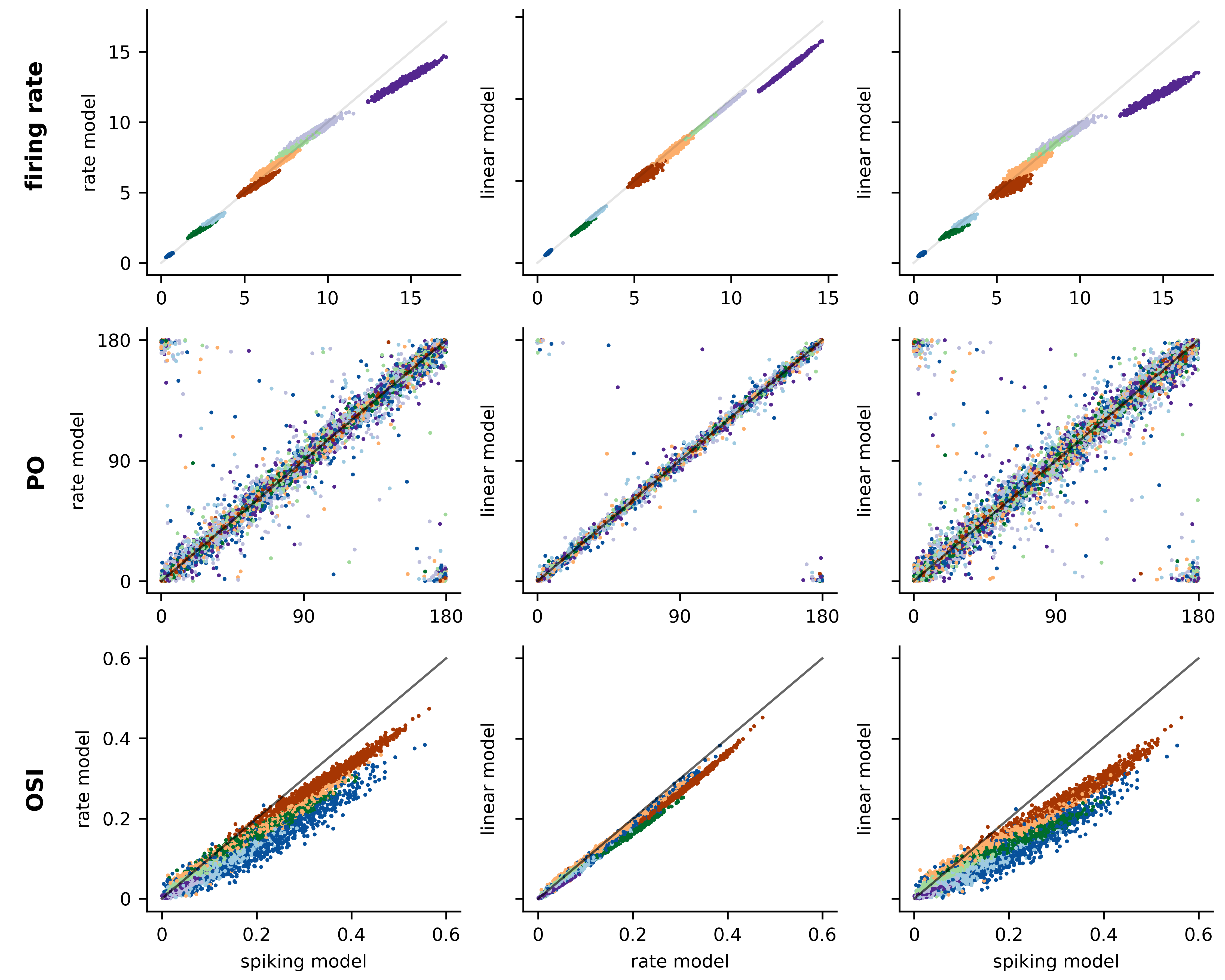

Supplement: S11 Fig — Comparison of the three central observables of the different network models. Top row: single neuron firing rates, middle row: preferred orientation (PO), bottom row: orientation selectivity index (OSI). The first column compares the spiking network model (Model A) with the firing rate model (Model B), the second column compares the firing rate model with linearized network model (Model C) and the third column compares the spiking network model with the linearized network model. Same color code is used as in previous figures to indicate the eight different populations. (TIF) [file pcbi.1007080.s011.tif]
